# Microbiological Assessment of Tap Water Following the 2016 Louisiana Flooding

**DOI:** 10.3390/ijerph17041273

**Published:** 2020-02-17

**Authors:** Nati K. Phan, Samendra P. Sherchan

**Affiliations:** Department of Global Environmental Health Sciences, School of Public Health and Tropical Medicine, Tulane University, New Orleans, LA 70118, USA; nphan@tulane.edu

**Keywords:** floodwater, fecal contamination, *E. coli*, fecal indicator bacteria

## Abstract

Floods are a prominent risk factor in the world of public health, as there is a risk of dispersal of harmful biological and chemical contaminants in floodwater. As climate change increases, the occurrence of natural disasters and risk of adverse health outcomes due to flash flooding also increases. Fecal indicator bacteria, such as *Escherichia coli* and *Enterococci,* are often encountered in contaminated floodwater and can cause gastrointestinal illnesses as well as a variety of infections. In August 2016, East Baton Rouge and surrounding parishes in Louisiana suffered heavy floods due to intense rainfall. No study of water quality during flooding has been conducted previously in Baton Rouge, Louisiana. Twenty-three pre-flush and post-flush water samples were collected immediately from accessible homes that had been affected by the floods in order to quantify concentrations of fecal indicator bacteria. These samples were analyzed for the presence of *E. coli* and *Enterococci* through both quantitative polymerase chain reaction (qPCR) and the IDEXX enzyme substrate method. The qPCR results indicated that 30% of the samples contained *Enterococci* and 61% of the samples contained *E. coli*, with the highest concentrations found in the pre-flush outdoor hose and the pre-flush kitchen tap. The IDEXX method yielded total coliforms in 65% of the samples, *E. coli* in 4%, and *Enterococci* in 35%, with the highest concentrations in the pre-flush outdoor faucet and the pre-flush post-filtration kitchen tap. Physical parameters including temperature, barometer pressure, dissolved oxygen, oxidation reduction potential, pH, conductivity, and salinity of these samples were also recorded. Of these parameters, conductivity and salinity were significant, suggesting they may positively influence *E. coli* and *Enterococci* growth.

## 1. Introduction

In the world of public health, floods and other natural disasters are especially significant events. Among all-natural disasters, floods are the most common after hurricanes, however they can affect a larger area and population than any other natural disaster [1]. Recent studies have revealed that floods can have unforeseen impacts on the environment as well as on public health. These impacts include mobilization of previously deposited hazardous contaminants and dissemination of previously contained microbes. According to the Centers for Disease Control, microbial contamination caused by flooding also poses a significant risk to affected populations [2]. Exposure to potentially pathogenic microbes occurs when flooding affects “wastewater treatment plants, residential septic systems, municipal sanitary sewer systems, and agricultural operations” [2]. As water treatment plants rely on electric power and tend to be located near rivers, water distribution systems are particularly at risk for adverse floods impacts, which can affect populations that use these systems [3].

Urban flooding can significantly affect the microbial loads and pose potential exposure risks to harmful pathogens. Some of recent flooding events include Hurricane Katrina and Hurricane Harvey of 2017 and the 2019 floods throughout the Midwest due to heavy rainfall [4,5,6]. Flooding is one of the most well-known events for contributing to water contamination in urban areas. Additionally, flooded homes might be hotspots for the proliferation of pathogens and antibiotic resistant bacteria [7]. However, there is lack of data on the dynamics of harmful pathogens and fecal indicator bacteria in urban floodwaters, particularly due to difficulty in sampling following the aftermath of such a disaster [8,9]. According to Paterson et al. [10], floods (excluding landslides) affected >74 million persons globally, resulted in 4720 deaths and had an economic cost of >$57 billion in 2016 alone. Therefore, floods are the most frequent natural disaster and can have widespread adverse health impacts [11]. It is imperative to understand microbial water quality after the flood and each flood disaster represents an opportunity for emergency preparedness and preventive measures to improve. In terms of water quality monitoring and regulation, fecal contamination to waterbodies is still assessed by enumerating fecal indicator bacteria (FIB) such as *Escherichia coli* (*E. coli*) and *Enterococci* in recreational waters [12].

When quantifying fecal bacteria from environmental sources, there are a range of methods available, however the two most frequently used are the IDEXX enzyme substrate method and the real-time quantitative polymerase chain reaction (qPCR) method [13]. Cultural methods are relatively cheap and easy to use; however, they require 18–24 h of incubation to produce results [14]. This can be an issue, as water quality notifications based on these methods then become delayed. Some other limitations of culture-based methods include inability to recognize viable but non-cultivable bacteria (VBNC) and low-growing bacteria, as well as possibility of cultivated organisms surviving and regrowing in the sediment after being released into environment [12,15,16]. On the other hand, qPCR methods can provide results in 3–4 h, which allows water quality notifications to be administered on the same day [12,14]. Molecular methods provide rapid, highly sensitive, and specific results, and they do not require cultivation or additional steps for confirmation [17]. However, the qPCR process is more complex and requires technical staff members with vigorous lab training and a high skill level. Furthermore, qPCR requires a skilled technician, dedicated lab space as well as certain reagents to prevent sample contamination from external DNA, as contamination can easily occur [17]. Other limitations of qPCR include detection of dead or non-viable cells, low amplification rates due to inhibitor substances, and lack of information on cell physiological activity [12,14,17]. A study by Whiley and Taylor [13] analyzed publications that used culture and qPCR to detect *Legionella* spp. from the last decade and found that 26/28 studies found higher levels of *Legionella* when using qPCR. Another study by Fatemeh et al. [15] compared PCR with the Most Probable Number (MPN) method and recommended PCR be used as an initial screening test, and positive samples should then be randomly tested by MPN.

In August of 2016, heavy rainfall led to floods that devastated parts of East Baton Rouge and other nearby parishes over a span of three days in Louisiana, USA. The levels of rainfall were over three times as high as the normal historical total precipitation averages for August, with some areas experiencing over 25 inches of rain [18]. As downstream conditions in the river caused upstream flooding, floodwaters were slow to subside, taking 10 days to return to normal levels [18]. Additionally, 13 victims lost their lives due to the flooding, through both direct and indirect effects of the flood. The range of health impacts resulting from a flood are broad, as they include infected wounds, injury complications, poisoning, poor mental health, communicable diseases, and starvation. All the fatalities resulting from the floods were due to some of these health impacts. Specifically, *E. coli* and *Enterococci* can cause gastroenteritis, skin infections, and eye infections [9]. No study of water quality during flooding has been conducted previously in affected areas around Baton Rouge, Louisiana. This is the first study aimed at determining the concentration of fecal indicator bacteria in drinking water following the 2016 flooding in Baton Rouge, Louisiana using both IDEXX and qPCR methods.

## 2. Materials and Methods

### 2.1. Sample Collection

Twenty-three 1 L water samples were collected immediately from different accessible houses at the flood affected areas 8 outside (O), 6 inside the kitchen (K), 2 from the sink tap, 3 from pressure tanks (PT), and 4 post-filtration from the kitchen tap (PFK). Pre-flush and post-flush samples were both collected and kept on ice immediately. Post-flush samples were collected after flushing the water for 3 min. Water samples were transported to the laboratory immediately for analysis. Physical and chemical water quality parameters, such as pH, temperature, dissolved oxygen, salinity, and specific conductance were measured in situ by using YSI Pro2030 Meter (Yellow Springs, OH, USA).

### 2.2. Fecal Bacteria Enumeration

Microbial analyses were performed for *E. coli* and *Enterococci* immediately upon returning to the laboratory via the IDEXX Colilert and Enterolert [19]. Defined Substrate Technology^®^ tests, respectively (www.idexx.com, IDEXX Laboratories, Westbrook, ME, USA). Briefly, 100 mL of water sample was each mixed with reagent and placed in Quanti-Tray/2000 then sealed using Quanti-Tray Sealer according to the manufacturer’s instructions. After incubation, the wells that fluoresced under ultraviolet (UV) light were quantified as positive for *E. coli* (IDEXX Colilert) and *Enterococci* (IDEXX Enterolert). The number of positive wells were compared to the manufacturer provided MPN table to enumerate *E. coli* and *Enterococci* in terms of MPN/100 mL [19].

### 2.3. DNA Extraction from Water Samples

On the day of sample arrival, each sample was filtered through 0.45 μm pore-size, 90 mm diameter nitrocellulose membranes (MF™ Millipore Membrane Filters, Merck KGaA, Darmstadt, Germany). After filtration, sterile forceps were used to aseptically fold each of the membrane filters and place them in separate sterile Petri dish plate and stored at −20 °C until DNA extraction. Genomic DNA was isolated from membrane filters using the PowerSoil^®^ DNA Isolation Kit (Mo Bio Laboratories, Inc., Carlsbad, CA) according to the manufacturer’s instructions. To maximize DNA extraction efficiency, membrane filters were cut into small pieces with sterile scissors and the DNA was quantified with a NanoDrop ND-2000 UV spectrophotometer(Thermo Scientific, Wilmington, USA). The DNA samples were stored at −20 °C prior to use.

### 2.4. Quantitative PCR Assays

qPCR assays targeting fecal indicator bacteria *E. coli* and *Enterococci* was used. The *E. coli* qPCR assay targeted an 83 bp fragment of the single copy *uidA* gene that codes for the enzyme β-D-glucuronidase and *Enterococci* qPCR assay targeted a 90 bp fragment of *23S rRNA* gene [12,19]. All qPCR assays were performed using the Applied Biosystems StepOne Real-Time PCR system (Applied Biosystems, NY, USA). The R^2^ values for all calibration equations were ≥0.99. The reaction mixture (20 µL) contained 1× PerfeCTa qCPR ToughMix (Quanta Biosciences, Beverly, MA), 0.2 µM of each primer, and 2.5 µL of the template DNA. qPCR reactions for *E.coli* were performed in duplicate and amplification protocols consisted of a hold at 95 °C for 3 min, followed by 45 cycles of 60 °C for 10 s and 72 °C for 10 s); and for *Enterococci,* it consisted of a hold at 95 °C for 2 min, followed by 40 cycles of 95 °C for 15 s, 60 °C for 60 s. Quantitative DNA standards were obtained from ATCC by using commercial genomic DNA from *E. coli* (ATCC 700926DQ^TM^, Manassas, VA, USA) and *Enterococci* (ATCC 29212Q-FZ, Manassas, VA, USA). A calibration curve with concentrations spanning the range from 10^1^ to 10^6^ gene copies per reaction, with two replicates, was prepared. Duplicate no-template controls (NTC) were included in each run. The amplification efficiencies (AE) were calculated based on the equation: AE = 10^(−1/slope)^ − 1.

### 2.5. Data Analyses

Statistical analyses were performed using R software. Spearman rank order correlation coefficient (*r*) was calculated to illustrate the relationships between FIB concentrations and physical parameters.

## 3. Results

### 3.1. Quantification of Total Coliforms and *E. coli* and *Enterococcus* Using IDEXX Method

**Figure 1:**
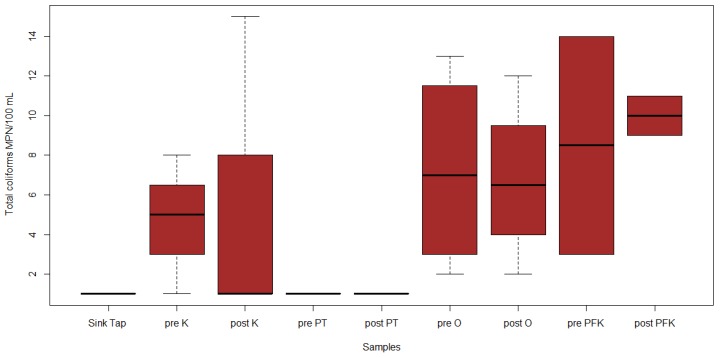
IDEXX total coliform concentrations (MPN/100 mL) in collected samples (outside (O), inside kitchen (K), pressure tanks (PT), and post-filtration kitchen tap (PFK).

**Figure 2:**
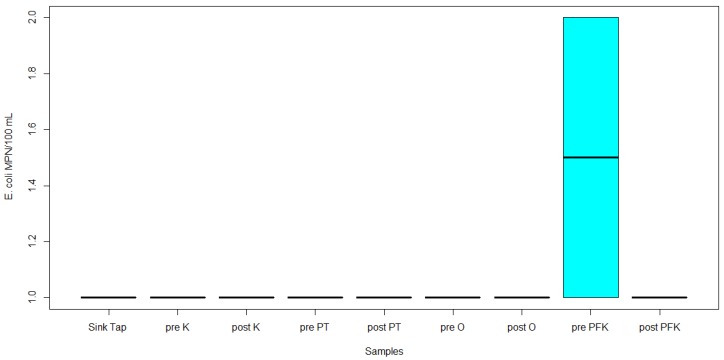
IDEXX *Escherichia coli* MPN/100 mL concentrations in collected samples (outside (O), inside kitchen (K), pressure tanks (PT), and post-filtration kitchen tap (PFK).

**Figure 3:**
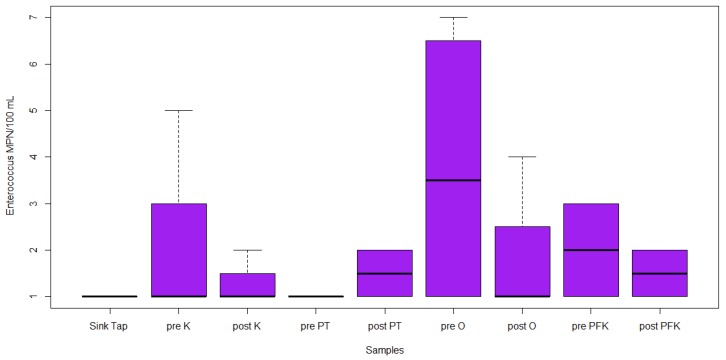
IDEXX *Enterococci* MPN/100 mL concentrations in collected samples (outside (O), inside kitchen (K), pressure tanks (PT), and post-filtration kitchen tap (PFK).

Twenty-three water samples were collected for the assessment of the microbiological quality of drinking water. Through the IDEXX cultural method, total coliforms were detected in 15/23 samples (65%), *E. coli* in 1/23 samples (4%), and *Enterococci* in 8/23 samples (35%). Out of the 23 samples, 2 pre-flush outdoor hose, 2 pre-flush outdoor faucet, 2 post-flush outdoor hose, 2 post-flush outdoor faucet, 2 pre-flush post-filtration kitchen tap, 2 post-flush post-filtration kitchen tap, 2 pre-flush kitchen tap, and post-flush kitchen tap (Figure 1) were positive for total coliforms. The highest concentration of total coliforms was 2450 MPN/100 mL in the pre-flush outdoor faucet. The lowest concentration of total coliforms was 1 MPN/100 mL in the pre-flush outdoor hose and the post-flush outdoor hose. However, there was no significant difference between these samples (*p* = 0.537, Figure 1). The one sample positive for *E. coli* was the pre-flush post-filtration kitchen tap, with a concentration of 2 MPN/100 mL (*p* = 0.212, Figure 2). Eight of the samples were positive for *Enterococci,* including pre-flush pressure tank, pre-flush outdoor hose, pre-flush outdoor faucet, post-flush outdoor faucet, pre-flush post-filtration kitchen tap, post-flush post-filtration kitchen tap, pre-flush kitchen tap, and post-flush kitchen tap (Figure 3). The highest concentration of *Enterococci* was 12 MPN/100 mL in the pre-flush post-filtration kitchen tap. The lowest concentration of *Enterococci* was 1 MPN/100mL in pre-flush pressure tank, post-flush post-filtration kitchen tap, and post-flush kitchen tap. However, there was no significant difference between these samples (*p* = 0.753, Figure 3).

### 3.2. qPCR Results of *E. coli* and *Enterococci*

**Figure 4:**
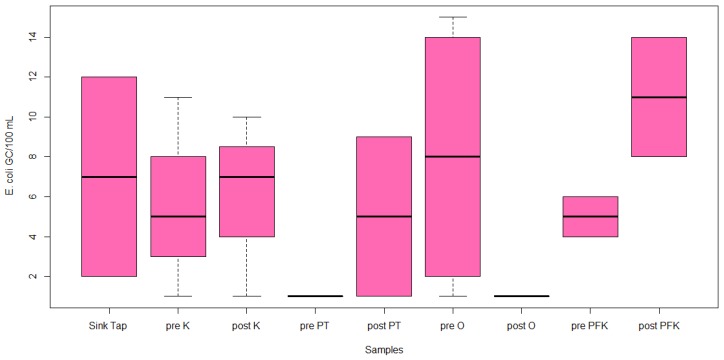
Distribution of *E. coli* (gene copies/100 mL) by qPCR (outside (O), inside kitchen (K), pressure tanks (PT), and post-filtration kitchen tap (PFK).

**Figure 5:**
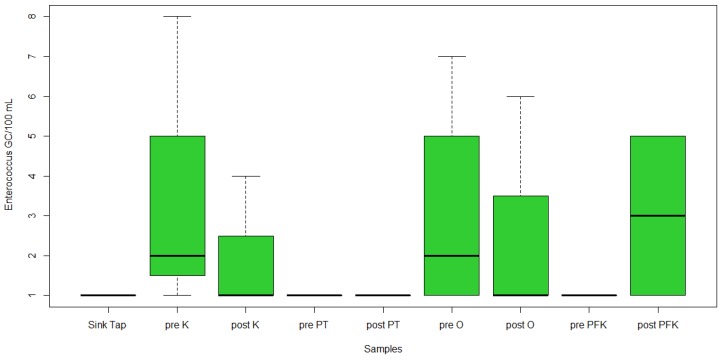
Distribution of *Enterococci* gene copies/100 mL by qPCR (outside (O), inside kitchen (K), pressure tanks (PT), and post-filtration kitchen tap (PFK).

*E. coli* DNA was detected in 14/23 samples (61%) and *Enterococci* in 7/23 samples (30%). Of the qPCR positive samples, *E. coli* was detected in the range between 1.8 and 1.9 log10 gene copies/100 mL, whereas *Enterococci* concentration was from 1.0 to 2.1 log10 gene copies/100 mL. Out of the 23 samples, 2 sink tap, 2 pre-flush kitchen tap, 2 post-flush kitchen tap, post-flush pressure tank, 2 pre-flush outdoor hose, 2 pre-flush post-filtration kitchen tap, 2 post-flush post-filtration kitchen tap, and pre-flush outdoor faucet (Figure 4) were positive for *E. coli*. The highest concentration of *E. coli* was 2.0 log10 gene copies/100 mL in the pre-flush outdoor hose. The lowest concentration was 1.8 log10 gene copies/100 mL in pre-flush kitchen tap, post-flush kitchen tap, pre-flush outdoor hose, pre-flush post-filtration kitchen tap, and post-flush post-filtration kitchen tap. However, there was no significant difference between these samples (*p* = 0.486, Figure 4). Out of the qPCR positive samples, *Enterococci* was detected in seven samples including 2 pre-flush kitchen tap, 2 pre-flush outdoor faucet, post-flush post-filtration kitchen tap, post-flush outdoor faucet, and post-flush kitchen tap (Figure 5). The highest concentration was 2.1 log10 gene copies/100 mL in the pre-flush kitchen tap. The lowest concentration was 1.0 log10 gene copies/100 mL in pre-flush kitchen tap and pre-flush outdoor faucet. However, there was no significant difference between these samples (*p* = 0.89, Figure 5).

### 3.3. Physical Parameters

The YSI Pro2030 Meter indicated the water sample temperatures ranged from 17.60 to 39.00 °C, with an average temperature of 22.75 °C (Table 1). The highest temperature of 39 °C was in pre-flush outdoor, and the lowest temperature of 17.60 °C was in the post-flush post-filtration kitchen tap. The dissolved oxygen in the samples had an average of 16.986 mg/L and ranged from 1.510 to 296.0 mg/L the highest being in the post-flush pressure tank (Table 1). The pH within the water samples had a minimum of 8.8 in post-flush outdoor and a maximum of 10.0 in the sink tap, while the mean pH was 9.38 (Table 1). The minimum oxidation reduction potential (ORP) was −219.0 mV in the post-flush outdoor, and the maximum ORP was −9.10 mV in the pre-flush outdoor, with an average of −79.71 mV (Table 1). The conductivity ranged from a low of 334.0 mS in the post-flush kitchen tap to a high of 721.0 mS in pre-flush outdoor, and the average conductivity was 489.5 mS (Table 1). The salinity of the water samples ranged from 0.17 to 0.30 ppt, with an average of 0.2478 (Table 1). All of the results for the physical parameters of the water samples were non-significant except for conductivity (*p* = 0.00088) and salinity (*p* = 5.29E−06). This indicates that an average conductivity of 489.5 mS and an average salinity of 0.2478 ppt are favorable for *E. coli* and *Enterococci* growth.

### 3.4. Correlations

Spearman’s correlational coefficient (r) was calculated for *E. coli, Enterococci,* and physical parameters to analyze the relationships between physical parameters and these bacteria. Total coliform MPN concentration was found positively correlated with *E. coli* gene copies/100 mL, *E. coli* MPN/100 mL, *Enterococci* gene copies/100 mL, *Enterococci* MPN/100 mL, ORP, conductivity (r = 0.467), and salinity (r = 0.461). Total coliform concentration was negatively correlated with temperature, barometer pressure, dissolved oxygen, and pH. *E. coli* gene copies/100 mL was positively correlated with *E. coli* MPN/100 mL, *Enterococci* MPN/100 mL (r = 0.315), dissolved oxygen, and ORP (r = 0.420). *E. coli* gene copies/100 mL was negatively correlated with *Enterococci* gene copies/100 mL, temperature, barometer pressure, pH, conductivity, and salinity. *E. coli* MPN/100 mL was positively correlated with *Enterococci* MPN/100 mL, dissolved oxygen, pH, ORP, conductivity (r = 0.321), and salinity (r = 0.308). *E. coli* MPN/100 mL was negatively correlated with *Enterococci* gene copies/100 mL, temperature, and barometer pressure. *Enterococci* gene copies/100 mL was positively correlated with *Enterococci* MPN/100 mL, pH (r = 0.120), ORP (r = 0.195), and salinity. *Enterococci* gene copies/100 mL was negatively correlated with temperature, barometer pressure, dissolved oxygen, and conductivity. *Enterococci* MPN/100 mL was positively correlated with ORP, conductivity (r = 0.116), and salinity (r = 0.258). *Enterococci* MPN/100 mL was negatively correlated with temperature, barometer pressure, dissolved oxygen, and pH (Table 2).

## 4. Discussion

In terms of environmental and public health, flooding is a well-known risk factor [9]. This study aimed to quantify the concentration of fecal indicator bacteria, *E. coli* and *Enterococci*, in water after the Louisiana flooding in 2016 using two different enumeration methods: (i) IDEXX enzyme-based and (ii) qPCR DNA-based methods. Twenty-three water samples were collected from different accessible houses in areas that had been impacted by major flooding in Louisiana. The original hypothesis indicated that flooding would cause fecal contamination in these water sources. However, when we began collecting water samples, it was a challenge to collect a lot of samples due to inaccessibility of homes during and following the flooding. Through the IDEXX method, total coliforms were detected in 65% of the samples, *E. coli* in 4%, and *Enterococci* in 35%. On the other hand, the qPCR method detected *E. coli* in 61% of samples and *Enterococci* in 30%. The large disparity between the IDEXX and qPCR result for *E.coli* detection may be explained by the inability of the IDEXX method to detect VBNC and low-growing bacteria in combination with the high sensitivity of qPCR and its ability to detect dead or non-viable cells [14,15]. Previous studies that have compared qPCR and cultural methods for detection of *Enterococci* have generally found that qPCR yields consistently higher numbers when compared to cultural methods [20,21]. A study by Noble et al. [22] compared *E. coli* detection between qPCR and Colilert and found a 94% agreement between the results of these two methods. This study also compared *Enterococci* detection between qPCR and IDEXX Enterolert, which yielded an 87% agreement rate [22]. As different environmental conditions may influence the prevalence of dead or nonviable cells, it is important to use both qPCR and cultural methods to obtain more accurate results.

The United States Environmental Protection Agency (USEPA)’s recreational water recommendation for *E. coli* and *Enterococci* are geometric means of 126 colony forming units (CFU)/100 mL and 33 CFU/100 mL [23]. The maximum *E. coli* and *Enterococci* concentrations obtained in this study were 2 MPN/100 mL and 12 MPN/100 mL, which fall below the EPA recreational standards. However, for drinking water, the WHO recommends that *E. coli* and total coliforms should be <1 in any 100 mL sample [24]. Additionally, the European Union (EU) does not permit *Enterococci* in any 100 mL water sample originating from a tap [25]. Although the results from the IDEXX method in this study yielded maximum concentrations under the recreational water standards, out of the 23 samples total coliforms were detected in 15, *E. coli* in one, and *Enterococci* in eight, which are above the WHO recommendation for drinking water. Isaac and Sherchan [12] also found *E. coli* in 1.5% of the tested drinking water samples in Louisiana using qPCR. Exum et al. [26] report that Federal Emergency Management Agency (FEMA) distributing more than 72 million liters of bottled water and nearly 17 million gallons of potable water during the Hurricane Maria response. Residents did not have trust and confidence in the quality of the water supplied by the public water system following Hurricane Maria and flooding events.

In this study, physical parameters of the water samples including temperature, barometer pressure, dissolved oxygen, ORP, pH, conductivity, and salinity, were also measured. Excluding conductivity (*p* = 0.00088, Table 1) and salinity (*p* = 5.29E−06, Table 1), all were non-significant. This suggests conductivity and salinity may have a positive effect on *E. coli* and *Enterococci* growth. When pathogens are deposited on different surfaces after a flood, drying will usually cause death or deactivation, however, under the right conditions, bacteria can survive for months [27]. *E. coli* can survive anywhere between 1.5 h and 16 months, and *Enterococci* can survive between five days and four months [28]. A study on recovery of *E. coli* found cell counts of *E. coli* decreased when exposed to UV radiation, however, salinity and temperature conditions in water were able to positively influence reactivation of these cells [29].

A study in Kentucky also measured *E. coli* and *Enterococci* with the IDEXX cultural method after flooding in the Ohio river [30]. In this study, Yard et al. [30] found that across 13 sampling sites during the floods there was a geometric mean 285 MPN/100 mL of *E. coli* and 335 MPN/100 mL of *Enterococci.* In the eight sample sites post-floods, there was a geometric mean of 13 MPN/100 mL of *E. coli* and 30 MPN/100 mL of *Enterococci*, these results suggest floods cause microbial contaminants in surface water to increase [30]. This study also measured pH, temperature, dissolved oxygen, and conductivity, all of which except pH were found to be significant (*p* < 0.01); whereas, in our study only conductivity and salinity were found to be significant.

A study in New Orleans after Hurricane Katrina found mean fecal coliform concentrations of 1.9E05 and 1.4E05 MPN/100 mL in Lakeview and Mid-City surface waters and mean concentrations of *E. coli* and *Enterococci* being 6.0E03 and 1.7E02 CFU/100 mL, respectively in Lake Pontchartrain [31,32]. These averages were much higher than our maximum *E. coli* and *Enterococci* concentrations of 2 and 12 MPN/100 mL, however as we collected samples from homes rather than from flood pools, canals, and lakes, this is expected. Additionally, a study by Yu et al. [9] in Houston done after the Hurricane Harvey floods also measured the presence of *E. coli* and *Enterococci* through the qPCR method. The highest concentration of *E. coli* was found to be 4.0 log10 gene copies/mL in indoor stagnant floodwaters [9]. Another study done in Houston after Hurricane Harvey sampled water from areas that had been affected by the floods. There, through qPCR, they found average concentrations of 2.1 log10 copies/100mL of *E. coli* and 5.7 log10 copies/100 mL of *Enterococci* [33]. Both studies found higher concentrations than our maximum *E. coli* and *Enterococci* concentrations of 1.9 log10 gene copies/100 mL and 2.1 log10 gene copies/100 mL.

During the 2012 Beijing flooding, Sun et al. [34] found the impact of flash flooding in drinking water quality. Nine water samples were collected and analyzed for eight parameters, namely turbidity, total hardness, total dissolved solids, sulfates, chlorides, nitrates, total bacterial count, and total coliform groups. Most samples exceeded China’s recommended thresholds. Islam et al. [35], during the 2004 flooding in Bangladesh found unacceptable levels of contamination of total coliforms (TC), fecal coliforms (FC), and fecal *Streptococci* (FS) respectively. In another study by Luby et al. [36], three flood-prone areas in Bangladesh were assessed for microbial quality and they found that tube well water samples were contaminated with total coliforms (41%, n = 85), thermotolerant coliforms (29%, n = 60), and *E. coli* (13%, n = 27). Man et al. [37] reported that urban floodwater contains pathogens including noroviruses, *Giardia*, *Cryptosporidium*. Khan et al. [38] collected 10 drinking water samples in Pakistan after flooding and revealed that twenty percent of the samples were contaminated with total fecal coliforms, *Salmonella*, *Shigella*, and *Staphylococcus aureus*, while forty percent of the samples were contaminated with *Vibrio*. Chaturongkasumrit et al. [39] collected 30 samples of floodwaters, river water, tap water, and filtered tap water from different sources following the flood crisis in Thailand in 2011. Eleven out of 12 samples exceeded the Thai bacterial water quality standards. All of these studies depict that it is paramount to monitor water quality after a flooding disaster [40,41]. In our study, we observed similar findings, through the IDEXX method, total coliforms were detected in 65% (15/23) of the samples, *E. coli* in (1/23) 4%, and *Enterococci* in (8/23) 35%. On the other hand, the qPCR method detected *E. coli* in (14/23) 61% of samples and *Enterococci* in (7/23) 30%.

## 5. Limitations

There are some limitations in this study. First, the sample size is not large. However, following the aftermath of such a disaster, it was very difficult to access flooded homes and get approval from the homeowners. Secondly, there is scarcity of data from coastal areas in Louisiana and only indicator bacteria were enumerated. Therefore, future study is needed in drawing general conclusions regarding the persistence of enteric pathogens in urban flood waters.

## 6. Conclusions

The results of this study demonstrate how flooding events can adversely impact drinking water by elevating levels of FIB. Both molecular and enzyme-based methods are used to quantify FIB, though qPCR tends to yield higher results due to its high sensitivity. Our results found *E. coli* in 61% of samples and *Enterococci* in 30% through the qPCR method, and through the IDEXX cultural method, total coliforms were detected in 65% of the samples, *E. coli* in 4%, and *Enterococci* in 35%. Out of the 23 samples, the pre-flush outdoor faucet, pre-flush post-filtration kitchen tap, pre-flush outdoor hose, and pre-flush kitchen tap were positive. Of the physical parameters measured, only conductivity and salinity were significant. *E. coli* and *Enterococci* may be removed from water through a variety of methods including exposure to UV light, chlorination, boiling, and electrocoagulation [40]. In lieu of a flood event, local governments should monitor water quality in drinking water distribution systems and administer boil water advisories if necessary.

## Figures and Tables

**Table 1:** Distribution of physical parameters by sample type (outside (O), inside kitchen (K), pressure tanks (PT), and post-filtration kitchen tap (PFK).

Sample Description	Total Coliform (MPN/100 mL)	*E. coli* (GC/100 mL)	*E. coli* (MPN/100 mL)	*Enterococcus* (GC/100 mL)	*Enterococcus* (MPN/100 mL)	Temperature (°C)	Barometer Pressure (mmHg)
Sink Tap	<1	75.5	<1	/	<1	23.1	759.7
pre K	<1	65.1	<1	117.5	<1	23.6	759.6
post K	<1	/	<1	/	<1	22.8	759.6
pre K	2	/	<1	9.6	<1	25.7	759.1
post K	<1	68.6	<1	/	<1	24.1	759.1
Sink Tap	<1	60.4	<1	/	<1	23.6	760.6
pre PT	<1	/	<1	/	<1	23.6	760.9
post PT	<1	69.8	<1	/	1	22.6	760.9
post PT	<1	/	<1	/	<1	23.1	760.9
pre O	1	61	<1	/	6.1	22.5	766.5
post O	1	/	<1	/	<1	21.7	766.7
pre O	8.1	90.5	<1	/	<1	39	766.6
post O	19.3	/	<1	/	<1	23.7	766.7
pre O	2450	/	<1	9.5	<1	20.8	759.6
post O	1986.3	/	<1	/	<1	21.2	759.7
pre PFK	101.7	67.5	<1	/	<1	22.4	758.4
post PFK	44.8	88.5	<1	24.3	<1	20.2	758.5
pre O	1413.6	79.9	<1	18.6	6.3	17.6	758.4
post O	727	/	<1	42	2	19.1	758.4
pre PFK	866.4	63.7	2	/	12	20.5	758.4
post PFK	235.9	69.5	<1	/	1	19.7	758.5
pre K	1732.9	75	<1	/	4.1	21.9	758.3
post K	920.8	71.5	<1	19.2	1	20.8	758.4

MPN: Most Probable Number. GC: genome copies.

**Table 2:** Correlation among fecal indicator bacteria and physical parameters.

	Total Coliforms MPN/100 mL	*E. coli* GC	*E. coli* MPN	*Enterococcus* GC	*Enterococcus* MPN	Temperature (°C)	Barometer (mmHg)	DO (mg/L)	pH	ORP (mV)	Conductivity (mS)	Salinity (ppt)
Total coliforms MPN/100 mL	1.000	0.171	0.328	0.460	0.310	−0.478	−0.408	−0.204	−0.362	0.224	0.467	0.461
*E. coli* GC/100 mL		1.000	0.000	−0.014	0.315	−0.141	−0.334	0.048	−0.099	0.420	−0.089	−0.034
*E. coli* MPN/100 mL			1.000	−0.138	0.265	−0.225	−0.259	0.064	0.000	0.193	0.321	0.308
*Enterococcus* GC/100 mL				1.000	0.093	−0.313	−0.425	−0.179	0.120	0.195	−0.117	0.094
*Enterococcus* MPN/100 mL					1.000	−0.594	−0.461	−0.057	−0.169	0.115	0.116	0.257
Temperature (°C)						1.000	0.563	0.090	0.020	−0.342	−0.239	−0.593
Barometer (mmHg)							1.000	−0.140	−0.191	0.173	0.126	−0.111
DO (mg/L)								1.000	0.105	0.024	−0.079	−0.041
pH									1.000	−0.437	−0.437	−0.206
ORP (mV)										1.000	0.145	0.099
Conductivity (mS)											1.000	0.870
Salinity (ppt)												1.000

MPN: Most Probable Number. GC: Genome copies. DO: Dissolved Oxygen. ORP: Oxidation Reduction Potential.
